# A Four-Gene Signature from Blood to Exclude Bacterial Etiology of Lower Respiratory Tract Infection in Adults

**DOI:** 10.21203/rs.3.rs-6033997/v1

**Published:** 2025-02-27

**Authors:** Ann Falsey, Derick Peterson, Edward Walsh, Andrea Baran, Chin-Yi Chu, Angela Branche, Daniel Croft, Micheal Peasley, Anthony Corbett, John Ashton, Thomas Mariani

**Affiliations:** University of Rochester; University of Rochester School of Medicine; University of Rochester; University of Rochester; University of Rochester; University of Rochester; Univeristy of Rochester.edu; University of Rochester; University of Rochester; University of Rochester; University of Rochester

## Abstract

Unnecessary antibiotic use is a major driver of antimicrobial resistance, an urgent public health threat. There is an unmet need for improved diagnostics for identifying bacterial etiology in acute respiratory infection (ARI). Hospitalized adults with ARI underwent comprehensive microbiologic testing and those with definitive viral (n = 280), bacterial (n = 129), or mixed viral-bacterial infection (n = 95) had whole blood RNA sequencing. A hard-thresholded, mostly relaxed, LASSO-constrained logistic regression model was used to select a parsimonious gene set (*ITGB4, ITGA7, IFI27, FAM20A*) highly capable of discriminating any bacterial from nonbacterial infection (cross validated AUC = 0.90). The 4-gene signature was validated in two independent cohorts (AUC = 0.90–0.94). Thresholding the 4-gene risk score to yield 90% sensitivity to detect bacterial infection resulted in 71% specificity and 91% negative predictive value. This 4-gene signature defining the absence of bacterial ARI may supplement clinical judgement for management of antibiotics in ARI.

## INTRODUCTION

Acute respiratory infections (ARI) account for substantial morbidity and mortality in adults, and are a leading cause of antibiotic overuse.^1,2^ In most cases of ARI, the precise microbial etiology is unknown and antibiotics are administered empirically, often unnecessarily, in both inpatient and outpatient settings.^3–5^ Although polymerase chain reaction (PCR) testing allows rapid diagnosis of respiratory viruses, the impact on antibiotic prescription has been modest primarily due to concern about bacterial co-infection.^6–8^ Importantly, there is currently a need for additional, sensitive and specific diagnostics for bacterial lung infection. “Ruling out” bacterial respiratory infection with current diagnostics is extremely difficult, resulting in a default position of prescribing antibiotics to most patients hospitalized with presumed respiratory infection. Unnecessary antibiotic use is a major driver of increasing antimicrobial resistance, one of the most urgent threats to global public health and as such more accurate microbiologic diagnostics for ARI are critically needed.^9,10,11^

Transcriptomics represents a powerful approach for analysis of the host response during infection.^12^ Earlier studies indicate that viral and bacterial infections trigger specific host transcriptional patterns in blood, yielding unique “bio-signatures” that may discriminate viral from bacterial causes of infection.^13–20^ Importantly, mixed viral-bacterial infection must be categorized with bacterial infection since antibiotic therapy is warranted and additionally, predictive genes should ideally be limited in number in order to be adaptable to development of rapid commercial tests. Substantial progress has been made in defining the host response to pathogens of global significance as well as in certain clinical syndromes including sepsis and chronic infection.^21–25^ However, translation of this knowledge into improved diagnostic tools to support clinician decision-making in the management of respiratory infections remains limited.^23,26,27^ Therefore, adults hospitalized with acute respiratory illness were enrolled and underwent comprehensive microbiologic testing and were adjudicated as bacterial or nonbacterial infection followed by RNA transcriptional profiling to develop gene expression predictors that discriminate bacterial and nonbacterial illness.

## RESULTS

### Cohort Description

**Figure 1:**
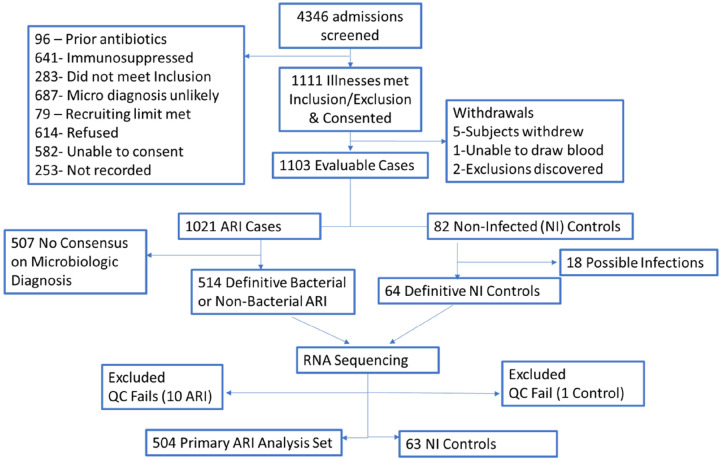
Consort Diagram.

Between March 2019 and April 2023, 4346 potential participants were screened for eligibility. The most common reasons for exclusion were immunosuppression (15%) and low likelihood to make a microbiologic diagnosis (16%) (Fig. 1). In addition, 14% refused participation and 13% could not provide consent, leaving 1111 enrolled of which 1103 were evaluable. Of the 1021 cases of ARI, 504 were adjudicated to have a definitive microbiologic diagnosis and underwent RNA sequencing and were included in the primary analysis. In addition, 82 cases were enrolled as non-infected control subjects of whom 64 were adjudicated as having sufficient microbiologic assessment to be classified as non-infected. The clinical characteristics of those included in the primary analysis of ARI compared to those without a definite microbiologic diagnosis who were not analyzed are shown in **Supplemental Table 1**. The primary analysis group was slightly younger and had significantly fewer chronic medical conditions, including history of smoking, COPD, home oxygen use and heart disease than the unanalyzed group. The analysis group required intensive care use more often but had lower rates of radiographic pneumonia. Finally, discharge diagnoses also differed with higher rates of bronchitis and viral syndrome in analyzed compared to the unanalyzed subjects, the latter who had higher rates of acute exacerbations of chronic obstructive pulmonary disease (AECOPD).

The analysis group was composed of 280 viral alone (V), 129 bacterial alone (B) and 95 mixed viral bacterial (VB) illnesses (**Details of each case are included in the supplementary materials**). The primary analysis compared cases with **any bacterial** illness (B and VB, N = 224) to cases with **nonbacterial** illnesses comprised of viral alone (V) illnesses (N = 280). The clinical features of each group are shown in Table 1. The any bacterial group was older with a higher percentage of smokers, more often had sputum production, exhibited confusion and more abnormalities of vital signs. Laboratory values such as total white blood cell count and serum procalcitonin were significantly higher and infiltrates on chest radiographs were more common in the any bacterial group. In addition, the any bacterial group had longer hospital stays and higher rates of intensive care and ventilatory support. Nonbacterial subjects were younger and more often had underlying asthma and upper respiratory infection symptoms. The discharge diagnoses were also significantly different between the two groups and aligned with the microbiologic category, with pneumonia and sepsis more frequent in the any bacterial group and asthma exacerbation and viral syndrome in the nonbacterial group. Of note, when comparing the VB subgroup to the B and V subgroups, the VB group most closely resembled the B group in terms of clinical presentation, laboratory parameters and outcomes, although there was a higher frequency of underlying asthma and URI symptoms at presentation (**Supplemental Table 2**).

The most frequent viral detections in the primary analysis group were rhinovirus (23%), influenza A (23%) and respiratory syncytial virus (10%) with few viral co-infections (4.1%) as shown in **Supplemental Table 3**. The most common bacterial pathogens detected were *Streptococcus pneumoniae* (12%), *Hemophilus influenzae* (7%), and Legionella (5%) and 2% had multiple bacterial detections. Of the viral-bacterial coinfections, the most common were influenza A and RSV with *Streptococcus pneumoniae* (9% each) and rhinovirus with *Hemophilus influenzae* (11%).

### Differential gene expression analysis of blood samples

**Figure 2:**
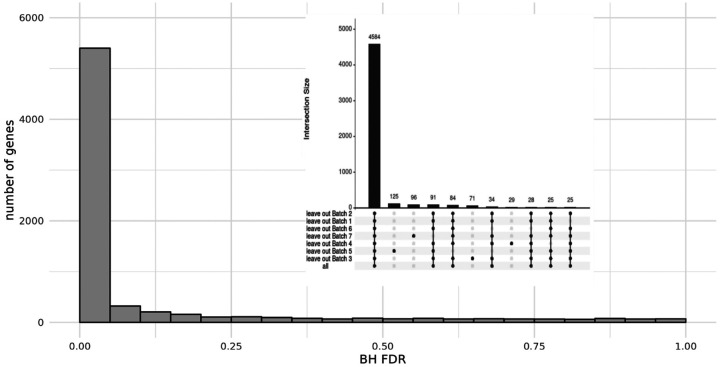
Differential Gene Expression. We identified 5401 differentially expressed genes (DEG) at an FDR<0.05 when comparing all subjects with a bacterial infection (B+BV, n=224) to those subjects with only a viral infection (V; n=280) using DESeq2. (***Inset***) We leveraged the existence of multiple sequencing runs (batches) in our cohort/data set to explore consistency in DEG identification, using a leave-one-batch-out (LOBO) approach (see Methods). Of the 5401 DEG found using all batches, 4584 genes (85%) were consistently found to be differentially expressed in all LOBO analyses.

Gene expression was compared between subjects with nonbacterial etiology (n = 280) and subjects with any bacterial infection (n = 224). On average 40 ± 11 million reads were generated from each of the cDNA libraries, with a mapping rate of 84.2 ± 1.1% and transcriptome coverage of 66.7 ± 10.8%. Differential expression analysis comparing the any bacterial to nonbacterial groups identified 5401 genes as significantly differentially expressed at FDR < 0.05. We leveraged the existence of multiple sequencing batches to explore consistency in differentially expressed genes by leaving out each of the 7 batches, one at a time. Of the 5401 genes identified using all batches, 4584 (85%) were consistently identified as differentially expressed when leaving out any one batch (Fig. 2).

### Diagnostic Feature Selection

**Figure 3:**
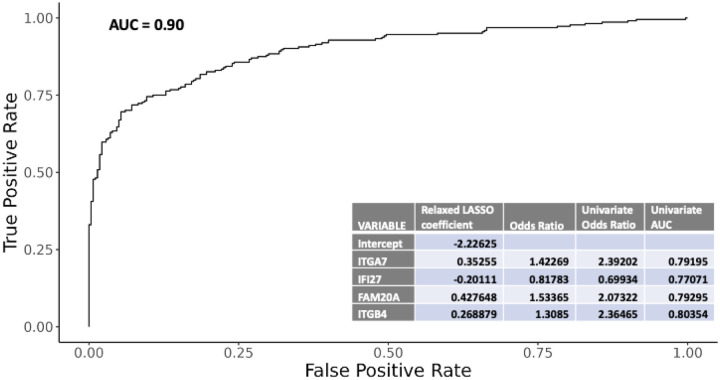
Diagnostic gene signature. A nested leave-one-batch-out cross-validation (CV) procedure tuning a hard-thresholded, mostly relaxed, LASSO-constrained logistic regression model was used to construct a parsimonious gene signature that distinguishes LRTI subjects with any bacterial infection from those with a viral only etiology (CV-AUC=0.90) (***Inset***) A table listing the names, adjusted contribution (relaxed LASSO coefficient/odds ratio), and univariate performance characteristics (univariate odds ratio and AUC) of the selected genes.

**Figure 4:**
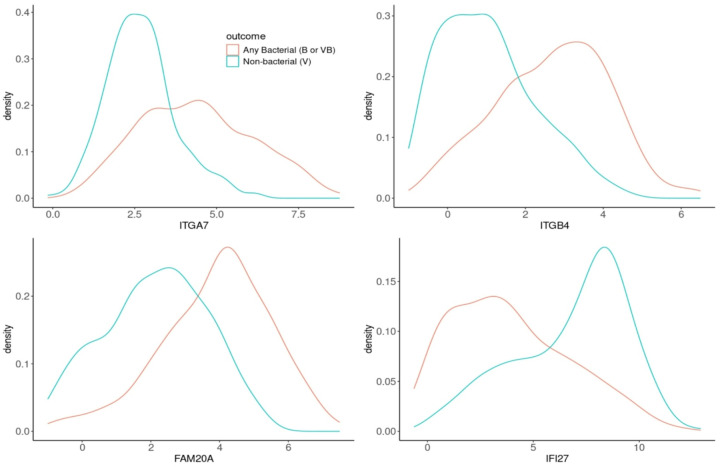
Expression profiles for the 4 genes in the signature. Distribution of gene expression for each of the 4 genes comprising the diagnostic gene signature by outcome. Any bacterial (B or VB) shown in red and nonbacterial (V) shown in blue.

We investigated the ability of gene expression patterns to discriminate any bacterial from nonbacterial infection. The cross-validation (CV) procedure relaxed LASSO parameters that resulted in a 4-gene diagnostic signature for any bacterial infection (Fig. 3). The final signature included weighted expression of ITGA7, IFI27, FAM20A and ITGB4. Nested CV was used to estimate performance of the procedure via receiver operating characteristic (ROC) analysis (CV-area under the curve [AUC] = 0.90) (Fig. 3). Density plots for the selected genes displayed separate, but overlapping expression patterns with *ITGA7*, *ITGB4* and *FAM20A* exhibiting higher expression in subjects with bacterial infection (Fig. 4) and the interferon-related gene *IFI27 exhibiting* higher expression in subjects lacking a bacterial infection. Interestingly, *ITGA7* and *ITGB4* expression levels were not distinctly different between bacterial subjects with (VB) or without viral co-infection (B), but *IFI27* and *FAM20A* showed clearly distinct patterns in the 3 underlying diagnosis groups (**Supplemental Fig. 1**).

### Validation of the 4-Gene Diagnostic Signature

We assessed the performance of our 4-gene signature using two external, independent data sets. First, we examined our own previously published cohort^17^ (phs001248.v1.p1) which consisted of 68 subjects (22 bacterial, 37 viral and 9 mixed bacterial-viral infections) recruited from the same Rochester, NY area population at an earlier epoch (January through June 2013). Our 4-gene signature discriminated between any bacterial and nonbacterial subjects with an AUC of 0.94 in this cohort (**Supplemental Fig. 2**). Next, we utilized data from a recent publication by Ko et al,^21^ (GSE211567) of 224 subjects (101 bacterial, 123 viral, co-infection status not defined) recruited from the US and Sri Lanka and our 4-gene model provided an AUC of 0.90 in this second cohort.

### Defining a threshold for classification

**Figure 5:**
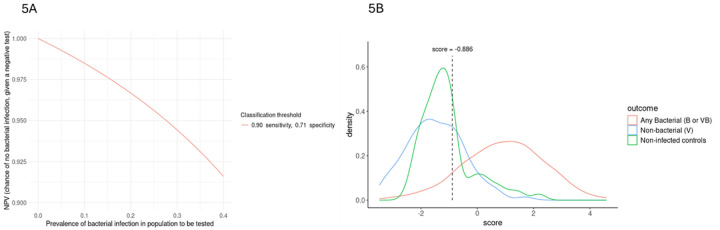
A gene signature-based diagnostic test. For practical implementation of a diagnostic test to exclude the involvement of bacterial etiology in LRTI, we aimed to find a risk score threshold that achieves both sensitivity and NPV >90%. (***5A***) The risk score threshold of −0.886 corresponds to 90% sensitivity and 71% specificity in the training data, and NPV ranges from 0.91 to 1.00 across a range of likely prevalences. (***5B***) The distribution of the 4-gene signature by outcome group shows good separation between bacterial and nonbacterial subjects. LRTI subjects with score ≥ −0.886 are classified as bacterial and subjects with scores < −0.886 are classified as nonbacterial. This threshold provides 75% specificity in the set of non-infected controls.

We sought to define a threshold score for the gene signature that can be used for classification of any bacterial infection. Based upon prior feedback from clinicians regarding the acceptability of a diagnostic test to exclude bacterial cause of LRTI, we aimed to achieve at least 90% sensitivity while also providing > 90% negative predictive value (NPV) for the determination of bacterial infection etiology in LRTI across a range of likely prevalences. The threshold corresponding to 90% sensitivity provided specificity of 71%, and NPV ranged from > 91% at 40% prevalence to > 95% at < 25% prevalence (Fig. 5A). This sensitivity/specificity combination corresponds to a gene signature threshold of −0.886, where LRTI subjects with score ≥ −0.886 would be classified as bacterial and subjects with scores < −0.886 are classified as nonbacterial.

Choosing this threshold to achieve 90% sensitivity resulted in 23 cases adjudicated as bacterial being classified as nonbacterial. Examining the 23 missed any bacterial infections using the 4-gene threshold score we found that 74% were VB and 74% were judged to be non-pneumonic ARI. (**Supplemental Table 4**) Of the 6 cases adjudicated as bacterial pneumonia that were scored as nonbacterial by our threshold, none were bacteremic or had consolidation on chest radiograph. The 4-gene signature also provided good separation of the noninfected controls from those with any bacterial infection. (Fig. 5B) Forty-seven (75%) of the non-infected controls were classified as nonbacterial using this threshold. Interestingly, a higher proportion of those misclassified as bacterial had a diagnosis of pulmonary embolism compared to those who were correctly classified as nonbacterial, 50% vs. 21%, p = 0.051, respectively.

## DISCUSSION

Bacterial antibiotic resistance has been identified as a major threat to global public health. Over 50% of hospitalized patients receive antibiotics and contact with health care facilities is a major risk factor for acquisition of antimicrobial resistant flora where these organisms flourish.^28^ Multiple studies have linked the incidence of colonization and subsequent infection with antibiotic resistant strains to previous antibiotic treatment.^29^ In 2013 the CDC estimated that approximately 2 million illnesses and 23,000 deaths occur yearly in the US due to antibiotic resistant bacterial infections.^11,30^ ARI are one of the most common reasons for emergency room visits and hospitalizations in the United States.^31^ The majority are treated with broad spectrum antibiotics, largely driven by diagnostic uncertainty regarding possible bacterial infection.^8,32^ Thus, management of hospitalized patients with ARI represents an opportunity to affect the induction and spread of antibiotic-resistant pathogens as well as reduce unnecessary drug related side effects.

Due to the inability to readily sample the lower airways via bronchoscopy or induced sputum for bacterial culture and difficulty clinically distinguishing any bacterial from nonbacterial causes of ARI, there has been growing interest in utilizing the host response to infection to predict the likelihood of bacterial infection. Serum biomarkers such as procalcitonin (PCT), C-reactive protein (CRP), TNF-related apoptosis inducing ligand (TRAIL) and Interleukin-10 have shown limited success as a supplement to clinical judgment in assessing patients with ARI, and currently biomarker testing is not routinely recommended for management of respiratory infections.^33,34^

In our study a number of traditional clinical features such as lack of URI symptoms, higher rates of sputum production, confusion and higher WBC and PCT levels were significantly different in participants adjudicated to have bacterial infection compared to viral alone. The panel of physicians had access to all clinical information and carefully reviewed all testing available suggesting that thorough evaluation of SOC data is useful in the clinical management of ARI. And yet, studies indicate that clinical parameters such as purulent sputum, WBC and radiographic patterns do not provide sufficient precision to reliably distinguish viral from bacterial infection.^35^ A recent systematic review found that symptoms and signs have a wide range and generally poor diagnostic accuracy for bacterial respiratory infection (sensitivity ranging from 9.6–89.1%; specificity ranging from 13.4–95%). Adding currently available biomarkers such as CRP and PCT improved sensitivity but was still not sufficient to be clinically useful, highlighting the need for a new approach.^33^ This corresponds with our findings that only half of cases enrolled in the study could be definitively clinically adjudicated as having a clear microbiologic classification, leaving the other half with significant diagnostic uncertainty.

Studies using whole blood gene expression indicate that viruses and bacteria trigger unique pathogen specific host “signatures” that can discriminate viral from bacterial causes of infection with greater accuracy than pct.^12,16,26,36,37^ Studies of adults and children with ARI and febrile illness have demonstrated relatively good accuracy of predictive gene sets (AUC ranging from 0.55 to 0.96 for bacterial classification). Interestingly, there has been little overlap in the predictive genes identified amongst the various studies.^38^ The difference in the predictive genes identified are likely explained by diverse populations, types of infection and control groups studied, along with alternate analytic tools used. Promising results have been reported from a number of studies of febrile illness or suspected sepsis. Rao et al. found that an 8-gene signature provided good accuracy to distinguish viral and bacterial infections using 69 data sets from 20 countries and Xu et al described a 2-gene classifier in febrile illness in Chinese children and adults.^22,23^ Most recently, a 29 gene-based predictor of bacterial sepsis, TriVerity^®^, which has an AUC of 0.83, has been approved for clinical use by the FDA based on a large clinical trial of patients with suspected sepsis.^39,40^ While very encouraging, most studies to date have not specifically focused on respiratory illness. In the largest study of respiratory infections to date, Tsalik et. al. used whole blood gene expression to discriminate bacterial from viral infection or non-infectious illness in 273 subjects with community onset ARI. Investigators defined 130 predictor genes in a model with an accuracy of 87% to discriminate clinically adjudicated bacterial, viral, and non-infectious illness.^15^ Recently a custom PCR test was designed to rapidly measure 45 host messenger RNA transcripts that could be performed on the automated BioFire Film Array platform.^41^ Even though a large number of host genes were required, this study provides proof of principle that a host gene signature can be translated to a clinical platform with timely results.

In our study, we sought to define a predictive gene expression signature capable of classifying nonbacterial ARI from ARI with any bacterial involvement, which could support a decision to withhold antibiotics. We identified a 4-gene set (*IFIT1, ITGA7, IFI27, FAM20A*) using nested cross-validation (CV) that was capable of discriminating any bacterial from nonbacterial infection with a CV-AUC of 0.90 specifically in adults hospitalized with cardiopulmonary illnesses. Its parsimony makes the 4-gene signature set an optimal candidate for translation to a clinically useful test to appropriately target antibiotic use for respiratory infections. Importantly, this 4-gene signature was validated in two independent cohorts, from which data had been processed differently and independently, with AUC of 0.94 and 0.90. Notably, one of the validation cohorts was a global study that included a variety of pathogens not included in the current study (dengue fever, leptospirosis and rickettsial diseases).^21^ In addition, our study included a robust sample of mixed viral-bacterial infections as part of the any bacterial infection group, 95 / 224 (42%). This very important subgroup of patients has not been well evaluated in prior studies. As antecedent viral infection is common in bacterial lung infection, this is a critical subgroup to include in developing predictors that will be clinically useful.^8^ Distinguishing viral from bacterial infection by relying on increased expression of interferon related genes alone could misclassify mixed viral-bacterial infections as viral only.

Of the four genes used in our classifier, only one (IFI27) is a canonical viral response, interferon related gene. *IFI27* encodes interferon alpha inducible protein 27, an important regulator of Interferon Stimulated Genes (ISGs) shown to counteract the innate immune response to avoid harmful overstimulation.^42^ *ISI27* has been included in other gene signatures for both viral and bacterial classification.^38,43^ Two of the other four classifier genes encode integrins (ITGB4 and ITGA7), membrane proteins that mediate a wide spectrum of cell to cell and cell to matrix interactions. *ITGB4* encodes instructions for synthesis of the β4 subunit of airway epithelial cell integrins, and has been found to be important in *in vivo* viral infections such as RSV, and in local inflammatory and immune responses.^44^ *ITGA7* encodes integrin subunit alpha 7, and has been studied as part of asthma related disease.^45,46^ Specifically, overexpression of ITGA7 has been associated with a decreased inflammatory response of the airways in mice but a phenotype of contractile airway smooth muscle cells in adults.^46^ In this way, overexpression of ITGA7 may decrease the inflammation needed to fight infection and also could lead to increased contractility in airway smooth muscle thereby increasing airway resistance and subsequent dyspnea. The final classifier gene *FAM20A* codes for a golgi localized type 2 transmembrane protein, with no clear role in infection responses. The mechanisms by which these 4 differentially expressed genes can accurately discriminate any bacterial from nonbacterial respiratory infections will require further study. However, their role, or lack thereof, in infection response pathophysiology does not preclude their diagnostic potential.

Thresholding the 4-gene risk score to yield 90% sensitivity resulted in 71% specificity to detect bacterial infection with a 91% negative predictive value at 40% prevalence of bacterial infection and > 95% at prevalence < 25%). In our study, the goal was to support decisions to withhold or withdraw antibiotics in hospitalized adults by developing an expression profile to exclude bacterial etiology, thereby prioritizing sensitivity over specificity to yield high NPV. In prior surveys we conducted amongst practitioners, only 8% would accept a bacterial misdiagnosis rate of ≥ 20%.^47^ Since the default position for most physicians has been to prescribe antibiotics “just to be safe”, especially for patients ill enough to warrant hospitalization, the specificity of 71% should still result in a substantial overall decrease in antibiotic use. In our prior study of ARI in hospitalized adults we found that 90% of patients judged to have viral infection alone received antibiotics during admission.^8^ Importantly, the cases of bacterial infection misclassified in the current study were primarily diagnoses of mixed viral-bacterial bronchitis suggesting minimal harm from using the gene expression score to guide antibiotic use. It is also possible that despite rigorous adjudication that some cases were not categorized correctly. Nevertheless, even accepting that no test is 100% accurate, this raises the possibility that gene expression could differ based on parenchymal vs mucosal infection. Thus, it may be worthwhile in the future to examine gene expression stratified by clinical syndrome of such as pneumonia versus non-pneumonic ARI.

Our study had a number of strengths but also some potential weaknesses. Strengths included the large sample size, inclusion of mixed viral-bacterial infections, comprehensive microbiologic evaluation and careful clinical adjudication by infectious diseases and pulmonary specialists. Potential weaknesses include dependence on clinical adjudication since a gold standard for bacterial respiratory infection does not exist and interruption of the study by the COVID-19 pandemic which changed clinical testing practices and de-emphasized bacterial sputum cultures. There were also differences in the characteristics of those analyzed compared to those not included. Due to the need for correct microbiologic designation to develop the predictors, cases that were analyzed were skewed to extremes of the clinical spectrum (ex. asthmatics with URI symptoms and wheezy bronchitis and persons with consolidative pneumonias). Thus, there may be value to evaluate the gene score in the remainder of the participants with less clear-cut diagnoses.

In conclusion, we developed a parsimonious gene set highly capable of discriminating any bacterial from nonbacterial infection. Our 4-gene signature yielded 90% sensitivity to detect bacterial infection with 91% NPV and was validated in two independent cohorts. This 4-gene signature may offer clinicians treating ARI a tool to supplement clinical judgment regarding antibiotic management of these common infections. Further study will be required to test if physicians will accept such a tool and if antibiotic use can be safely reduced and produce improved outcomes for patients hospitalized with ARI.

## ABBREVIATED METHODS

Details of Methods are provided in the supplementary materials.

### Study Period and Sites

The study was conducted at two hospitals in Rochester, N.Y; University of Rochester Medical Center (URMC) and Rochester General Hospital (RGH) between March 2019 and April 2023. The study was approved by the relevant institutional review boards and all participants or their legally authorized representatives signed written informed consent prior to study procedures. Enrollment was paused from March to October 2020 due to the COVID19 pandemic.

### Recruitment

Potential participants with symptoms of an acute cardiopulmonary illness or diagnoses compatible with acute respiratory infection (ARI, i.e., pneumonia, acute exacerbation of chronic obstructive pulmonary disease, bronchitis, asthma, upper respiratory infection, influenza, viral syndrome) were screened by reviewing hospital admission logs. A limited number of patients with noninfectious cardiopulmonary diagnoses were included as control cases. Participants were enrolled within 24 hours of admission if hospitalized.

### Acute Illness Evaluation

At enrollment demographic, clinical and laboratory information were collected from the medical record and direct patient and family interviews. Medical history and medications, date of illness onset and signs and symptoms were collected. Results of standard of care (SOC) testing were recorded.

### Clinical and microbiological adjudication

Cases were adjudicated by a panel of four physicians (three infectious diseases and a pulmonary medicine specialist) and classified into discrete microbiologic categories of viral infection alone, bacterial infection alone or bacterial-viral coinfection. Confidence in the microbiological classification was rated as definitive, probable, or indeterminate, and required unanimous agreement by adjudicators. Only cases judged as definitive were included in the primary analysis and blood samples sent for RNA sequencing. Noninfected (NI) control cases required a clear noninfectious event with a negative NS PCR to exclude asymptomatic viral infection. Controls were contacted 7–14 days later to ensure their non infected status had not changed.

### Laboratory Methods

Whole blood was collected in Tempus^™^ Blood RNA Tubes and RNA isolated using the Tempus Spin RNA Isolation Kit (Applied BioSystems). Total RNA was processed for globin reduction using GLOBINclear Human Kit and cDNA library construction was performed using the TruSeq Stranded mRNA library kit (Illumina, San Diego, CA) as described previously.^48^ Libraries were sequenced on the Illumina NovaSeq6000 (Illumina, San Diego, CA). Reads were mapped to the Human GRCh38/genecode38 reference using STAR, counts were summarized with HTSeq^49^ and counts per million (CPM) normalized.^50^ Gene-specific mean CPM values were used to filter genes with insufficient expression (mean CPM < 2) for downstream analyses. Principle component analysis (PCA) was used to identify and remove 11 outlier samples (10 ARI and 1 NI). Sequencing run (batch)-specific variance modeling was used to remove additional genes. After subject and gene filtering, we retained 504 ARI samples and 7,352 genes for downstream analyses.

#### Statistical Methods:

A leave-one-batch-out nested cross-validation procedure was used to tune a hard-thresholded, mostly relaxed, LASSO-constrained logistic regression model to construct a gene signature to discriminate any bacterial (B and VB) from nonbacterial (V) infections. Independent validation of the gene signature was performed in two datasets with comparable populations and clinical outcomes: Kho et. al.^21^ (GSE211567) and Bhattacharya et. al.^17^ (phs001248.v1.p1). For both independent validation datasets, we applied the coefficients from the final gene signature to construct a risk score for each validation subject. An ROC curve with associated AUC was used to assess the performance of the risk scores in each independent validation set.

To derive a classifier from the gene signature, we identified the threshold that provided ≥ 90% sensitivity, which also yielded 91% NPV across a range of likely prevalences (≤ 40%) of bacterial infection. Any subject with a gene signature ≥ threshold is classified as bacterial, and any subject with a gene signature < threshold is classified as nonbacterial.

Clinical variables were compared between subjects with any bacterial infection vs. nonbacterial infection using t-tests for continuous variables or Fisher’s exact tests for categorical variables. P-values are two-sided, with p ≤ 0.05 indicating nominal statistical significance. Differences in expression between any bacterial and nonbacterial ARI subjects for each gene were assessed using DESeq2 with FDR< 0.05.

## Figures and Tables

**Table 1:** Study Populations and Illness Characteristics of Primary Analysis Cases

Characteristic	Bacterial * N = 224	Nonbacterial** N = 280	P value
**Mean Age (SD)**	63.1 ±15.9	59.8 ± 18.4	**0.03**
**Female, No (%)**	105 (47)	176 (63)	**0.0004**
**Race, No (%)**			0.051
White	166 (74)	181 (65)	
Black/AA	55 (25)	90 (32)	
Other	3 (1.3)	9 (3)	
**Hispanic**	17 (8)	36 (13)	0.059
**Medical Conditions**			
Mean BMI (SD)	31.5 ± 10.1	32.3 ± 9.8	0.369
Asthma	61 (27)	128 (46)	**< 0.0001**
COPD	82 (37)	83 (30)	0.105
Any Smoking	177 (79)	196 (70)	**0.025**
Home Oxygen	26 (10)	30 (11)	0.777
CAD	34 (15)	53 (19)	0.288
CHF	32 (14)	33 (12)	0.425
Diabetes Mellitus	68 (30)	84 (30)	1.0
Chronic Kidney Disease	27 (12)	29 (10)	0.571
Any medical condition	218 (97)	276 (99)	0.351
Mean No. Medical Conditions (SD)	3.8 ±1.8	3.8 ±1.8	1.0
**Symptoms & Signs**			
Nasal Congestion	109 (49)	195 (70)	**< 0.0001**
Sore throat	66 (29)	138 (49)	**< 0.0001**
Cough	209 (93)	271 (97	0.091
Dyspnea	208 (93)	253 (90)	0.340
Sputum production	171 (76)	174 (62)	**0.0007**
Feverish	153 (68)	170 (61)	0.093
Confusion	22 (10)	12 (4)	**0.019**
Highest pulse	108 ± 23.5	105 ± 18.3	0.083
Lowest Systolic BP	116 ± 20.5	125 ± 26.3	**< 0.0001**
Respiratory rate	26 ± 8.1	24 ± 7.1	**0.003**
Oxygen Saturation	90 ± 7.1	91.2 ± 6.8	**0.054**
Temperature	37.9 ± 1.0	37.5 ± 0.90	**< 0.0001**
**Laboratory**			
WBC	14.7 ± 7.7	8.8 ± 3.8	**< 0.0001**
BUN	12.0 ± 4.4	17.0 ± 11	**< 0.0001**
PCT	6.9 ± 18.6	0.13 ± 0.15	**< 0.0001**
CXR - any infiltrate	120 (54)	46 (16)	**< 0.0001**
**Hospital Course**			
ICU	53 (24)	22 (8)	**< 0.0001**
Non-invasive Ventilation	27 (12)	22 (8)	0.131
Mechanical Ventilation	12 (5)	1 (0.4)	**0.0004**
In hospital death	5 (2)	2 (0.7)	0.250
**Primary Discharge Diagnoses**			
Asthma	9 (4)	40 (14)	**0.0001**
Bronchitis	26 (12)	19 (7)	0.083
AECOPD	28 (13)	54 (19)	0.052
Pneumonia	84 (38)	16 (6)	**0.0001**
Respiratory Failure	12 (5)	15 (5)	1.0
Viral Syndrome	13 (6)	76 (27)	**< 0.0001**
Sepsis	14 (6)	0	**< 0.0001**
Other	38 (17)	60 (21)	0.215

* Bacterial includes 129 bacterial alone and 95 mixed viral bacterial cases.

** Non-bacterial are viral alone cases.
